# Socioeconomic inequality in organized and opportunistic screening for gastric cancer: results from the Korean National Cancer Screening Survey 2009–2022

**DOI:** 10.3389/fpubh.2023.1256525

**Published:** 2023-10-09

**Authors:** Xuan Quy Luu, Kyeongmin Lee, Jae Kwan Jun, Mina Suh, Kui Son Choi

**Affiliations:** ^1^Department of Cancer Control and Population Health, Graduate School of Cancer Science and Policy, National Cancer Center, Goyang, Republic of Korea; ^2^National Cancer Control Institute, National Cancer Center, Goyang, Republic of Korea

**Keywords:** gastric cancer, Korea, socioeconomic inequality, organized screening, opportunistic screening

## Abstract

**Objectives:**

This study aimed to evaluate the socioeconomic inequality in gastric cancer (GC) screening in Korea. Socioeconomic inequality was assessed using both organized and opportunistic screening according to income and educational level.

**Methods:**

GC screening data were obtained from the 2009–2022 Korean National Cancer Screening Survey. The final analysis included 47,163 cancer-free men and women. The weighted cancer screening rate was estimated using joinpoint regression. The inequality indices were measured in terms of both the absolute slope index of inequality (SII) and the relative index of inequality (RII) using the Poisson regression model.

**Results:**

The organized screening rate for GC increased from 38.2% in 2009 to 70.8% in 2022, whereas the opportunistic screening rate decreased from 18.8 to 4.5%. Regarding educational inequality, a negative SII value was observed [−3.5, 95% confidence interval (CI), −7.63–0.83%] in organized screening, while a positive SII (9.30%; 95% CI, 6.69–11.91%) and RII (1.98%; 95% CI, 1.59–2.46) were observed in opportunistic screening. Furthermore, income inequality was not found in organized GC screening; however, overall SII and RII for opportunistic screening were 7.72% (95% CI, 5.39–10.5) and 1.61 (95% CI, 1.42–1.81), respectively.

**Conclusion:**

Organized screening rates have grown gradually over time and account for the majority of GC screenings in South Korea. While no socioeconomic inequalities were found in organized screening, significant socioeconomic inequalities were found in opportunistic screening.

## 1. Introduction

Gastric cancer (GC) has large regional variations worldwide (1). More than 60% of the incidence of GC cases occur in Eastern Asia, with an age-standardized rate (ASR) of 22.4 cases per 100,000 (1). This figure was much lower in Western countries and regions where the ASR was about 7 per 100,000 in Southern Europe, Central America, and only 4.6 in Northern Europe (1). The differences are due to the various socio- demographic characteristics, dietary behaviors, prevalence of *Helicobacter pylori* infection, and genetic factors (2–4). Although its incidence rate has recently been decreasing, GC remains the fourth leading cause of cancer-related deaths worldwide (1). In Korea, GC has been decreasing constantly, with an annual percentage change (APC) of −4.8; however, it still ranks as the fourth most common cancer, with 26,662 new GC cases in 2020 (an ASR of 25.7 cases per 100,000) according to the Korea Central Cancer Registry (KCCR) (5). Almost two-thirds of cancer cases were prevalent in men, and people aged between 60 and 69 years had the highest GC burden (6). In addition to regional differences in GC burden, significant inequalities in GC incidence and outcomes within the country have also been reported (7–9). Using data from Korea Central Cancer Registry, a study found that the ASR of GC was lower in the metropolitan areas (6). In the US, compared to non-Hispanic White people, other ethnic groups have a higher risk of GC and GC mortality; it could be up to 1.89 times higher among non-Hispanic Asian/Pacific Islanders for GC incidence and 2.6 times higher among non-Hispanic Black people for GC mortality (8). The uneven distribution of the GC burden can be explained by not only ethnic/regional factors but also an individual’s socioeconomic status (SES) and health related systems, such as accessibility to healthcare services, low health literacy, and financial difficulties (7, 10–12).

Some Asian countries have introduced GC screening programs (13–16). Organized screening programs are known to contribute not only to reducing the cancer burden but also to reducing inequality in disease burden (11, 12). However, inequality issues in cancer screening, wherein advantaged people had a higher participation rate (11, 12, 17, 18), have been reported. In Korea, the Korean National Cancer Screening Program (KNCSP) offers either an upper gastrointestinal series (UGIS) or upper endoscopy as the primary GC screening test for people aged ≥40 years biannually (19). GC screening, particularly endoscopy, has been demonstrated to have a positive impact on the survival of patients with GC and significantly decrease GC mortality (20–22). Since the introduction of GC screening in Korea, the overall GC screening rate, including organized and opportunistic screening, has steadily increased from 39.2% in 2004 to 72.8% in 2018 (23). Specifically, the participation rate of the KNCSP for GC increased significantly from 7.4% in 2002 to 62.9% in 2019 (24). As one of the main goals of the KNCSP is to eliminate socioeconomic inequality, some studies have been conducted to assess this aspect (18, 25, 26). However, these studies focused only on investigating the inequality in the overall screening rate (18, 26) or were conducted during the early phase of the KNCSP (25). Thus, an updated and comprehensive evaluation of inequalities in GC screening is required, particularly when comparing organized and opportunistic screening approaches.

Therefore, this study aims to evaluate the socioeconomic inequality in GC screening in Korea. Socioeconomic inequality was assessed in both organized screening and opportunistic screening according to income and educational level.

## 2. Materials and methods

### 2.1. Study material

This study used data from the Korean National Cancer Screening Survey (KNCSS), a national survey conducted annually since 2004, to investigate cancer screening behaviors among Korean men and women. The KNCSS was designed using a stratified multistage sampling method based on the area, age, and sex of the population. The study data were collected through face-to-face interviews that were conducted by a professional research agency. In case of any missing information, a trained staff member contacted the respondent via telephone to ensure the completeness of the records. After conducting the survey for over two decades, the study sample size has been adjusted and expanded to improve the survey quality and to reflect the change in the screening policy. During the early years of the KNCSS, the overall survey sample size was approximately 2,000 men aged 40–75 years and women aged 30–75 years, which was later increased to 4,100 in 2010 and 4,500 in 2014. The detailed sampling method for the KNCSS has been previously described (19, 23). The current study analyzed the KNCSS data from 2009 to 2022 to compare the annual trend of the screening rate according to socioeconomic factors. Since the main concern of the current study was GC screening, only individuals aged 40–75 were included according to the protocol of the KNCSP in Korea. The final dataset comprised 47,163 men and women aged 40–75 with no history of any type of cancer.

This study was approved by the Institutional Review Board of the National Cancer Center of Korea (IRB Number: NCC-2019-0233). All participants were informed of the purpose and use of the data before enrollment in the study, and the requirement for written informed consent was waived.

### 2.2. Measurements

According to the KNSCP protocol, both upper endoscopy and UGIS are recommended for examining and visualizing the entire upper GI tract every 2 years for men and women aged 40 years or above (23). During the upper endoscopy procedure, if any abnormal tissue (recorded as possible GC, early GC, advanced GC, or others where the physician deemed it necessary) is detected, a biopsy may also be performed for investigation. In the case of those who chose UGIS, if suspicious findings were observed, the patient was referred for follow-up procedure with upper endoscopy and biopsy for the final laboratory confirmation.

Recommendation-based GC screening was defined based on the question, “Have you ever undergone gastric cancer screening by [UGIS/upper endoscopy]?” and “When was the last screening round you had with this test method?” Individuals who underwent screening with the recommendation were defined as those who underwent screening by either UGIS or upper endoscopy within the past 2 years, according to the recommendation of GC screening in Korea. Furthermore, screening types were divided into organized and opportunistic groups using questions about the source of their payment. Organized screening is a systematic and planned program that targets a specific population. In Korea, the government introduced the KNCSP as an organized cancer screening program, and screening costs are largely covered by the National Health Insurance System (NHIS). Opportunistic screening is sporadic and occurs when individuals seek medical care or when healthcare providers offer screening tests based on an individual’s demographics, risk factors, or symptoms and generally require individuals to pay. Therefore, those who responded that the government or NHIS paid for GC screening were classified as organized screened, and those who reported that they paid for themselves were classified as opportunistic screened. The demographic characteristics included age, sex, and residential area. Socioeconomic factors included education level and household income. There were four subgroups based on the highest completed educational level: elementary school or no formal education, middle school, high school, and college/university or higher. Monthly household income contained three labels: low, middle, and high, which were divided based on the tertile distribution of the 13 original categories, ranging from approximately 1,000 USD to 10,000 USD or more. Owing to the change in the SES of the population over the study period, different cut-off points were applied for the original income categories to illustrate the income distribution of the target population. The household income was divided as follows: <2000, 2000–3,499, ≥3,500 for 2009; <2,500, 2,500–3,999, ≥4,000 from 2010 to 2012; <3,000, 3,000–4,499, ≥4,500 for 2013; <3,500, 3,500–4,499, ≥4,500 from 2014 to 2018; and < 3,500, 3,500–4,999, ≥5,000 from 2019 to 2022.

### 2.3. Statistical analysis

The weighted screening rate was reported by the type of screening (organized or opportunistic screening) for each survey year from 2009 to 2022. The trend in the screening rate was assessed using the joinpoint regression model. Based on the real pattern of the screening rate, the model produced the best-fit line(s), which could be either single or multiple segments. To summarize and compare the trends in screening rates, the average annual percentage change (AAPC) of GC screening rates over the past 14 years is reported.

Inequality indices are measured in both absolute and relative terms to obtain a comprehensive view of inequality. The absolute inequality was reported as the slope index of inequality (SII). The weighted sample for each year of the survey was ranked in consecutive order from the lowest education/income level to the highest education/income level. The weighted rankings accounted for the distribution of the target population in each subgroup. Subsequently, based on the cumulative proportion of the ranked socioeconomic variables and the midpoint of this socioeconomic variable, a new socioeconomic variable was generated and input into the Poisson regression model to estimate the regression coefficient (SII). A zero SII indicates no inequality; a positive SII reflects higher screening participation among advantaged people; and a negative value indicates the opposite. The relative index of inequality (RII) was estimated using the same procedure, in which RII is the ratio of the screening rate in the most privileged SES group to the least privileged SES group. A RII of one indicates no inequality. A RII value of >1 is interpreted as the fold change in the screening rate between the most privileged individuals and the least privileged individuals, and the opposite is true for an RII value of <1. The pooled SII and RII over 14 years of study were estimated using a random-effects meta-analysis model. The time trend was estimated by fitting the meta-regression model, wherein the survey year was treated as an independent variable. Further, a subgroup analysis was conducted to investigate the issue of inequality by sex and residential area.

Descriptive analyses and estimations of the SII and RII were performed using Stata version 16 (Stata Corp., College Station, TX, United States). The Joinpoint Regression Program, version 4.9.1.0 (Statistical Research and Applications Branch; National Cancer Institute, Rockville, MD, United States), was used for the trend analysis of the screening rate. Statistical significance was set at a *p-*value <0.05.

## 3. Results

**Figure 1:**
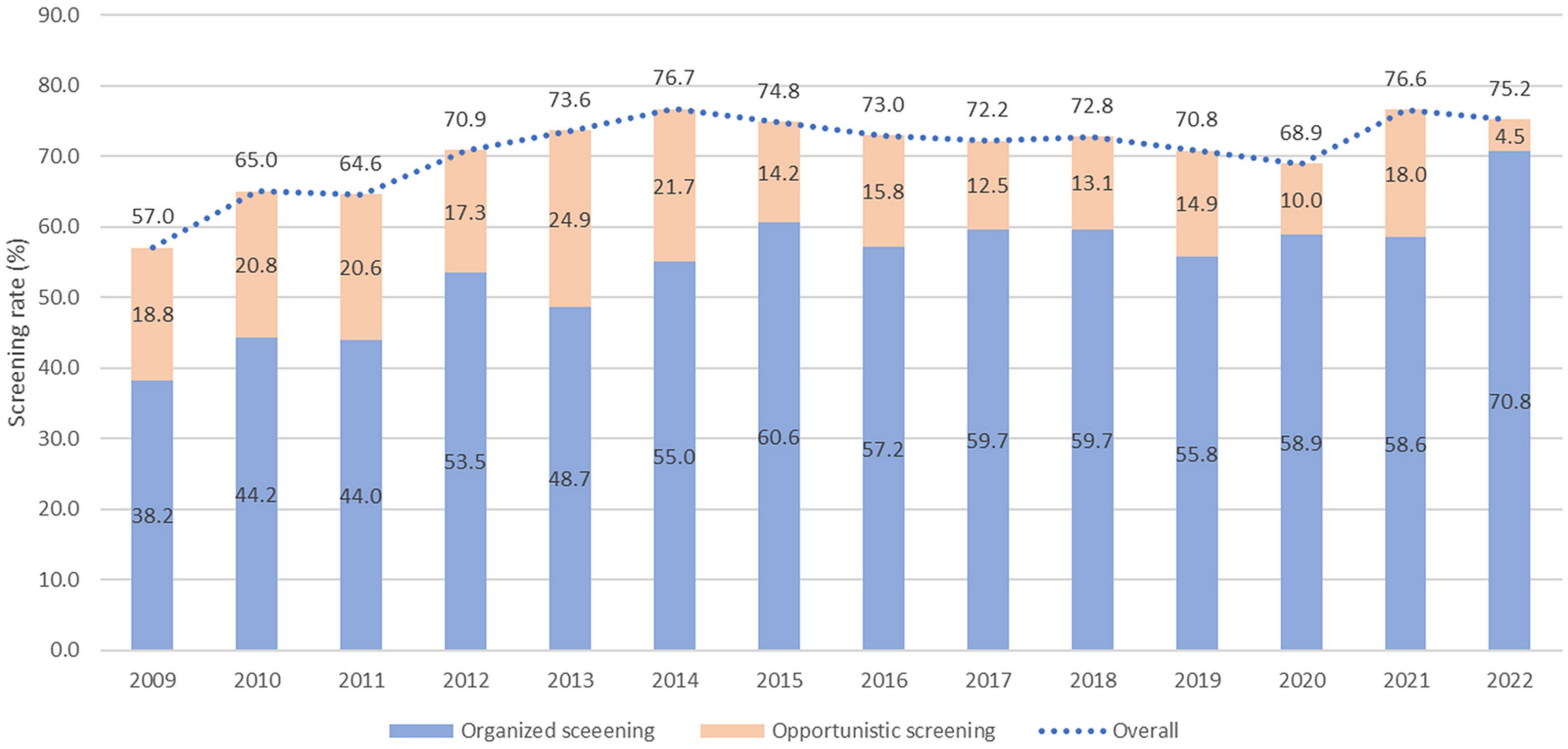
Gastric cancer screening rate by type of screening from 2009 to 2022.

Table 1 shows the weighted baseline characteristics of the KNCSS over 14 years of study. Except for the 2009 survey, most surveys included information from approximately 3,500 men and women eligible for GC screening. The unweighted baseline characteristics of the study are presented in Supplementary Table S1. The overall screening rate with the recommendation increased constantly from 57.0% in 2009 to more than 75.2% in 2022, where organized screening had a similar pattern and contributed to the majority of the GC screening rate (Figure 1). The GC screening rate rapidly increased in the period 2009–2014, with an APC of 4.81% (*p* < 0.001); however, it fluctuated in the subsequent years (Supplementary Figure S1). Supplementary Table S2 shows the overall screening rate by subgroup according to trend.

**Table 1:** Baseline characteristics of the study population in the Korean National Cancer Screening Survey, 2009–2022.

	2009	2010	2011	2012	2013	2014	2015	2016	2017	2018	2019	2020	2021	2022
Total, no.	1,640	3,411	3,474	3,498	3,509	3,441	3,441	3,480	3,484	3,495	3,539	3,647	3,552	3,552
Sex, %														
Male	49.1	49.5	49.6	49.6	49.5	49.7	49.8	49.8	49.8	49.8	49.4	49.4	49.9	49.9
Female	50.9	50.5	50.4	50.4	50.5	50.3	50.2	50.2	50.2	50.2	50.6	50.6	50.1	50.1
Age group, %														
40–49	42.9	41.0	39.9	39.9	38.6	38.0	37.2	36.3	35.5	34.8	31.5	30.6	31.7	31.6
50–59	30.6	32.2	33.8	33.8	34.8	34.7	34.6	34.6	34.6	34.5	32.4	31.8	33.1	33.1
60–69	19.5	19.5	19.1	18.9	19.6	19.7	20.7	21.7	22.5	23.3	22.9	24.0	27.0	27.2
70–75	7.1	7.3	7.3	7.4	7.0	7.7	7.6	7.3	7.3	7.4	13.2	13.5	8.2	8.1
Residential area, %														
Metropolitan	46.2	44.0	44.8	44.6	44.1	44.5	45.4	43.9	44.8	44.0	43.7	45.4	43.0	43.0
Non-metropolitan	53.8	56.0	55.2	55.4	55.9	55.5	54.6	56.1	55.2	56.0	56.3	54.6	57.0	57.0
Education level, %														
Elementary or lower	18.1	9.8	9.5	11.2	6.0	6.4	5.9	4.4	5.0	4.2	4.5	4.7	3.8	3.3
Middle school graduates	13.3	13.0	12.4	10.1	8.4	9.3	9.7	7.2	11.0	9.6	11.9	10.0	7.8	7.2
High school graduates	46.7	52.4	52.9	53.4	53.9	56.2	54.8	55.6	52.9	52.0	52.3	53.7	50.2	49.6
College/University or higher	21.9	24.8	25.1	25.3	31.7	28.0	29.7	32.7	31.0	34.3	31.3	31.7	38.2	39.9
Household income, %														
Low	28.9	32.6	32.3	27.6	25.4	36.9	37.6	37.8	38.3	35.5	34.9	36.5	30.2	25.3
Middle	45.1	36.2	36.5	44.9	34.6	26.5	27.4	30.2	31.7	25.9	37.5	32.5	32.6	28.6
High	26.0	31.2	31.2	27.5	40.0	36.6	35.0	32.0	30.0	38.5	27.7	31.0	37.1	46.1

The organized screening rate for GC has nearly doubled, increasing from 38.2% in 2009 to 70.2% in 2022, with an AAPC of 4.2 (*p* < 0.001) (Table 2). The sharpest increase in organized screening rate was observed between 2009 and 2015, with an APC of 6.7% (*p* < 0.001) (Supplementary Figure S1). A statistically significant increase in organized screening rates was observed in almost all the subgroups (Table 2). The opportunistic screening rate fluctuated between 2009 and 2014 and subsequently experienced a significant decrease to only 4.5% in 2022, with an AAPC of −4.9% (*p* < 0.001) (Table 3 and Supplementary Figure S1). The steepest decrease in the opportunistic screening rate was observed among older individuals, high school graduates, those with lower levels of education, and people with low household income (Table 3).

**Table 2:** Organized screening rates for gastric cancer according to socioeconomic status in the Korean National Cancer Screening Survey 2009–2022 (%).

	2009	2010	2011	2012	2013	2014	2015	2016	2017	2018	2019	2020	2021	2022	AAPC (95% CI)
Total	38.2	44.2	44.0	53.5	48.7	55.0	60.6	57.2	59.7	59.7	55.8	58.9	58.6	70.8	4.2 (1.2–7.4)
Sex															
Male	32.6	39.2	39.5	49.7	46.4	53.8	59.0	54.7	54.8	57.2	54.6	57.4	54.7	71.0	3.6 (2.1–5.2)
Female	43.6	49.2	48.4	57.3	50.9	56.2	62.2	59.6	64.5	62.1	57.0	60.4	62.5	70.5	2.4 (1.4–3.5)
Age group															
40–49	29.4	35.9	35.4	46.3	46.3	50.5	53.0	50.9	58.4	54.2	51.6	53.1	50.8	68.5	4.0. (2.3–5.8)
50–59	43.7	50.3	49.2	58.1	50.2	56.5	63.8	59.5	59.7	62.1	58.7	61.2	60.2	71.8	2.3 (1.2–3.4)
60–69	48.4	52.0	51.3	59.1	49.9	59.4	68.4	63.6	61.1	65.4	62.1	66.0	64.0	73.4	2.4 (1.3–3.4)
70–75	39.7	43.6	47.7	57.2	51.0	59.3	61.8	58.1	61.8	56.0	47.6	54.0	64.8	66.4	3.5 (−1.1–8.4)
Residential area															
Metropolitan	37.6	45.1	45.1	48.7	50.4	60.5	64.7	59.8	60.6	63.9	56.7	58.8	59.0	72.4	3.0 (1.7–7.8)
Non-metropolitan	38.8	43.5	43.1	57.4	47.4	50.6	57.2	55.1	58.9	56.3	55.1	59.0	58.3	69.6	2.9 (1.6–4.2)
Education level															
Elementary or lower	47.8	51.4	52.8	59.4	51.5	48.7	54.1	68.4	74.3	49.5	50.3	55.8	61.2	65.4	1.8 (−0.3–3.8)
Middle school graduates	46.8	50.7	50.1	58.7	52.9	53.9	62.6	64.9	58.1	65.7	45.9	60.2	63.5	67.2	1.8 (0.3–3.4)
High school graduates	34.6	44.5	44.2	55.1	50.1	56.7	63.2	58.6	59.7	60.7	59.0	62.2	63.4	70.1	3.0 (1.8–4.2)
College or higher	32.8	37.5	37.1	45.6	44.8	53.5	56.6	51.5	57.8	57.7	55.1	53.3	51.0	72.7	4.2 (2.3–6.1)
Household income															
Low	46.0	49.8	49.8	55.9	47.1	56.4	60.7	60.9	59.8	59.0	54.4	58.5	63.1	69.3	2.1 (1.0–3.2)
Middle	35.0	40.5	40.3	53.2	48.6	58.5	60.8	55.9	59.2	59.6	54.8	57.4	57.5	70.3	5.0 (0.5–9.7)
High	35.1	42.7	42.3	51.8	49.8	51.1	60.4	53.9	60.0	60.3	59.0	61.0	55.8	71.8	3.6 (2.3–4.9)

Screening with recommendation was defined as the upper gastrointestinal series, or upper endoscopy, during the past 2 years. AAPC, Average Annual Percent Change; 95% CI, 95% confidence interval.

**Table 3:** Opportunistic screening rates for gastric cancer according to socioeconomic status in the Korean National Cancer Screening Survey 2009–2022 (%).

	2009	2010	2011	2012	2013	2014	2015	2016	2017	2018	2019	2020	2021	2022	AAPC (95% CI)
Total	18.8	20.8	20.6	17.3	24.9	21.7	14.2	15.8	12.5	13.1	14.9	10.0	18.0	4.5	−4.9 (−8.6 to –1.1)
Sex															
Male	19.7	24.4	24.0	20.1	26.0	23.4	16.5	16.8	15.6	15.9	16.2	11.9	22.1	5.3	−4.3 (−7.8 to –0.7)
Female	17.9	17.2	17.2	14.6	23.8	20.0	12.0	14.9	9.3	10.4	13.7	8.2	14.0	3.6	−5.7 (−9.9 to –1.2)
Age group															
40–49	18.1	23.8	23.6	18.8	26.2	25.4	17.6	17.7	11.4	17.9	18.4	12.9	23.2	4.0	−3.3 (−7.4–0.9)
50–59	18.8	19.3	19.6	18.0	24.4	21.0	13.6	14.9	15.3	12.3	15.4	10.0	19.8	5.1	−4.0 (−7.8 to 0.1)
60–69	21.4	17.8	17.7	15.8	24.5	18.5	11.0	12.9	10.9	9.1	13.0	7.6	12.2	4.3	−7.2 (−10.9 to –3.4)
70–75	16.3	18.5	15.9	10.4	21.3	14.1	9.2	19.7	9.0	7.4	9.1	7.8	10.0	4.2	−7.5 (−11.9 to –2.9)
Residential area															
Metropolitan	17.4	19.9	19.3	20.7	23.9	18.1	10.3	15.7	12.6	12.5	16.3	9.5	18.2	4.8	−4.6 (−8.5 to –0.5)
Non-metropolitan	20.0	21.4	21.6	14.6	25.7	24.5	17.5	15.9	12.3	13.7	13.9	10.5	17.9	4.2	−5.3 (−9.3 to –1.1)
Education level															
Elementary or lower	15.8	14.0	12.5	10.9	23.5	21.9	9.4	11.0	7.4	2.8	5.5	5.2	10.4	0.9	−6.1 (−13.3 to 1.8)
Middle school graduates	17.5	14.4	14.1	14.1	17.0	21.0	12.0	15.2	8.9	7.5	10.3	3.8	8.3	1.5	−7.1 (−12.0 to –2.0)
High school graduates	17.9	18.8	18.7	15.9	24.3	20.6	12.8	14.2	11.0	11.8	13.2	8.4	12.5	3.3	−6.7 (−10.5 to –2.7)
College or higher	24.2	31.0	30.8	24.5	28.2	24.0	18.5	19.4	17.1	18.0	20.9	15.6	28.1	6.7	−3.9 (−7.7 to 0.1)
Household income															
Low	14.3	14.4	13.5	13.8	24.0	18.6	11.1	12.7	9.8	9.5	11.2	8.0	9.2	2.8	−6.7 (−11.3 to –1.8)
Middle	18.8	21.0	21.0	15.9	23.3	20.4	12.5	16.3	11.2	14.0	14.4	9.6	18.9	3.5	−3.6 (−7.7 to 0.8)
High	23.8	27.2	27.4	23.1	26.8	25.7	19.0	19.1	17.2	15.9	20.4	12.8	24.5	6.0	−3.9 (−8.2 to 0.6)

Screening with recommendation was defined as the upper gastrointestinal series, or upper endoscopy, during the past 2 years. AAPC, Average Annual Percent Change; 95% CI, 95% confidence interval.

**Figure 2:**
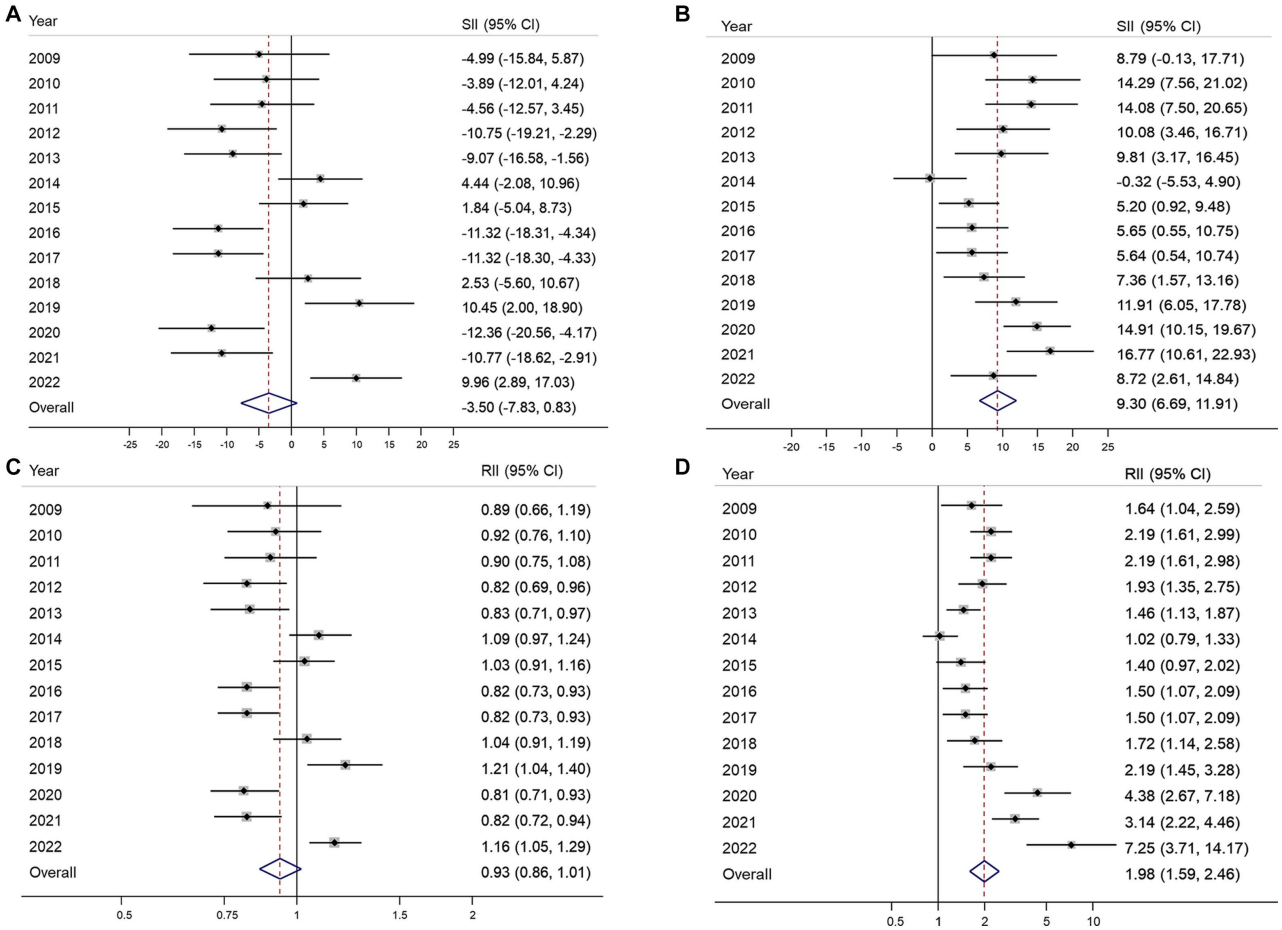
Absolute and relative educational inequalities in organized **(A,B)** and opportunistic **(C,D)** gastric cancer screening from 2009 to 2022. SII, absolute slope index of inequality; RII, relative index of inequality; 95% CI, 95% confidence interval.

Figure 2 illustrates the absolute and relative inequalities in terms of educational level for both organized and opportunistic GC screenings. Negative SII values were observed in eight of the 14 years of the study, while significant positive SII values were recorded only in 2019 and 2022 (Figure 2A). The pool estimate of SII for organized GC screening was −3.5% (95% CI, −7.63–0.83%). Similarly, the RII was mostly below 1, and no significant inequality was observed in the overall estimate of the index (RII 0.93; 95% CI, 0.86–1.01) (Figure 2B). In contrast, the SII for opportunistic screening remains significantly positive in almost all years of the survey except for 2009 and 2014, given the pooled SII of 9.3% (95% CI, 6.69–11.91%) for the whole period of 14 years (Figure 2C). The RII for educational inequality in opportunistic screening ranges from approximately 1 in 2014 to 7.25 in 2022. The overall RII was 1.98 (95% CI, 1.59–2.46), indicating that university graduates are about two times more likely to engage in opportunistic screening compared to those who completed elementary school or lower.

**Figure 3:**
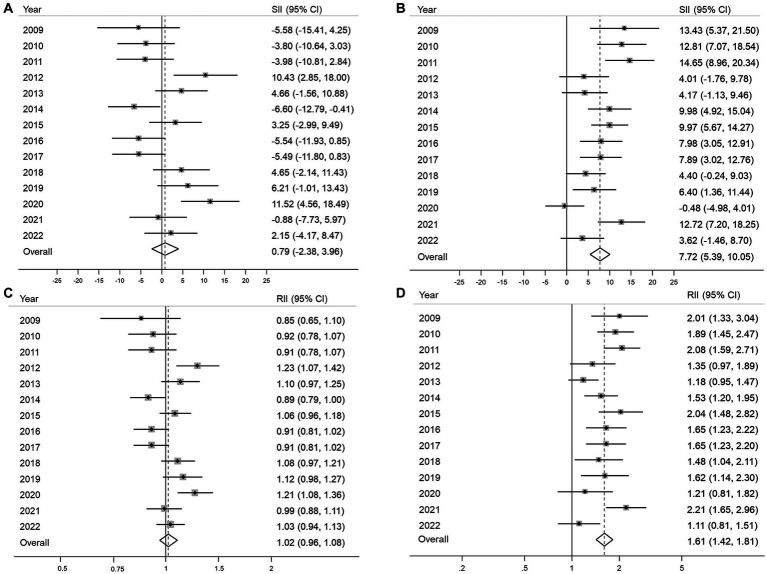
Absolute and relative income inequalities in organized **(A,B)** and opportunistic **(C,D)** gastric cancer screening from 2009 to 2022 SII, absolute slope index of inequality; RII, relative index of inequality; 95% CI, 95% confidence interval.

No significant income inequality in either absolute or relative terms was observed in 12 of the 14 years of the study period (Figures 3A,B). The overall SII and RII for income inequality in organized GC screening were 0.79% (95% CI, −2.38–3.96%) and 1.02 (95% CI, 0.96–1.08), respectively (Figure 3B). In contrast, the opportunistic screening showed significant income inequality with positive SII in most years, and the overall SII was 7.72% (95% CI, 5.39–10.5) (Figure 3C). The RII of income inequality ranged from 1.18 (95% CI, 0.95–1.47) in 2013 to 2.47 (95% CI, 1.28–4.79) (Figure 3D). Overall, the high-income group had 1.61 times (95% CI, 1.42–1.81) higher opportunistic screening rate compared to the low-income group (Figure 3D).

In the subgroup analysis by sex, educational inequality was more evident among men in both the absolute (men: SII of 12.3, 95% CI, 8.08–16.18; women: SII of 5.89, 95% CI, 2.85–8.93) and relative measures (men: RII of 2.22, 95% CI, 1.68–2.93; women: RII of 1.65, 95% CI, 1.27–2.14) of inequality for opportunistic screening (Supplementary Figures S2, S3). Notably, a significant increasing trend was observed in relative educational inequality for opportunistic screening in women (*p* for trend = 0.017). The pattern was relatively similar for income inequality between both sexes with no significant trend observed (Supplementary Figures S4, S5). For the organized screening method, no inequalities were found in the overall estimates for both education and income. By residential area type, there were no obvious differences between people living in metropolitan and nonmetropolitan areas; educational inequality was also in good agreement with the main analysis (Supplementary Figures S6, S7). There was a significant decreasing trend for income inequality in SII for opportunistic screening (*p* for trend = 0.033); however, no trend was observed in RII (Supplementary Figures S9C,D).

## 4. Discussion

This study indicated a noteworthy increase in the GC screening rate, primarily driven by higher participation in organized screening. In contrast, opportunistic screening experienced a significant decrease in the overall rate and among specific subgroups. While certain years of the study showed education and income inequalities, no socioeconomic inequality was observed in the overall estimates of both absolute and relative indices for organized GC screening. However, socioeconomic inequality was frequently observed in opportunistic screening throughout each year of the study as well as in the pooled estimates, suggesting that individuals with lower SES encounter barriers or inequalities in accessing and utilizing opportunistic screening for GC. However, these barriers or inequalities were not observed in organized screening.

Income and educational level have been well addressed as the main socioeconomic factors influencing participation in GC screening (27–29). Using data from the Korean National Health and Nutrition Examination Survey, Kwon et al. and Chang et al. reported significant differences in GC screening attendance, wherein higher educational qualifications or higher income were positively associated with GC screening (27, 29). An analysis of more than 15,000 Japanese women reported that those with a lower SES were less likely to participate in GC screening in urban areas (28). Lee et al. found socioeconomic inequality in GC screening for both education and income (18). Unfortunately, this study did not investigate the inequality by screening type. In the context of cancer screening, inequality is reportedly less serious in regions with organized screening than in countries without screening programs (30, 31). A nationwide survey in Korea reported that the educational inequality index in organized screening moved toward zero (no inequality) between 2005 and 2009, whereas inequality in opportunistic screening persisted and tended to increase (25), suggesting that organized screening programs play a positive role in reducing socioeconomic inequality during cancer screening, which is consistent with our findings.

In the current study, an organized screening program appeared to be more effective in achieving equitable utilization of GC screening across different socioeconomic groups, highlighting the importance of organized screening programs to reduce SES-related inequalities in GC screening. Organized screening, with its structured and systematic approach, may have been effective in reaching and engaging individuals of lower SES. Furthermore, alleviating the individual cost burden of GC screening by providing 100 or 90% of the screening costs in the public sector is believed to have significantly contributed to mitigating the inequality associated with the financial burden of screening. Opportunistic screening for GC primarily involves an upper endoscopic screening test, which is also included as a primary screening test in the KNCSP (13). Through the promotion and implementation of a screening invitation system, more individuals have become aware of the KNCSP. Additionally, the screening units in the KNCSP have extended their operating hours, offering services until 9 p.m. on specific days of the week and even opening on weekends. As a result, the overall participation rate in GC screening, particularly organized screening, has increased to over 70% in recent years.

Notably, the screening rate can also be influenced by external factors, such as the COVID-19 pandemic (32, 33). In 2021, when COVID-19 was serious and several social distancing policies had been implemented, the opportunistic screening rate had increased to 18% from approximately 10% in previous years. This increase can be attributed to individuals’ fear of contracting COVID-19 in crowded and high-risk areas such as hospitals or general clinics. Consequently, some individuals are willing to pay for opportunistic screenings. Since socioeconomic inequality is significantly associated with opportunistic screening participation, the rise in opportunistic screening participation during the COVID-19 pandemic could potentially lead to broader inequalities.

Based on our findings, we note that a well-organized screening program is essential for reducing the inequality in cancer screening and the overall cancer burden. While both opportunistic and organized screening facilities are available for the general population, policy makers should consider adopting/revising the appropriate screening policy to maximize participation in organized screening policies. Some insights that can be taken from the implementation of GC screening in Korea include the offering of high-quality screening tests, conducting mass media campaigns for screening, a comprehensive invitation/follow-up system, and individualized strategies for the lower SES groups (23, 25, 29). Similarly, for GC burden, Japan has been implementing a national cancer screening program since the 1980s. Currently, both radiographic and endoscopy tests are being recommended for GC screening in Japan. However, compared with Korea, the participation rate has remained low, and this relates to the different aspects of the guidelines and management system (34). There is a lack of regulation for quality assurance in screening programs in Japan (34). In contrast, the KNCSP’s quality assurance system is governed by law, and the results of all cancer screenings are collected and linked to other national databases such as the cancer registry and death certificates for the process of continuous monitoring and evaluation of the KNCSP (16, 19, 34). Further, as the issue of inequality is subjected to change by the internal and external factors of the screening program, continuous monitoring/evaluation of the program indicators and the inequality issue will help the program to have on-time action for a good quality screening program. The GC screening program contributes significantly toward improving the survival of GC cancer patients and eventually reducing the GC mortality rate (21, 22). Thus, the equal delivery of organized screening has a positive effect on the inequality in the GC cancer burden as well, where people, especially in the low SES group, have an equal opportunity to screen and detect cancer at the early stage with a much lower cost of treatment and a higher survival or cure rate.

The current study has some limitations. First, the use of survey data might impose some recall bias on the reported information. However, we used non-clinical information such as sociodemographic characteristics, which has been reported to have good accuracy (35, 36). For the screening history, when comparing the self-reported history and clinical records, the sensitivity ranged from 96.5% in Tsuruda et al.’s study and up to 100% in the results reported by Hoffmeister et al. (35) and Tsuruda et al. (36) Therefore, the use of self-reported GC screening information, especially within the last 2 years, is highly accurate in our study. Second, the use of SII and RII requires ordinal variables; therefore, only education and income levels were used in our analysis. Future studies should also consider including the other variables in sensitivity analysis for a more comprehensive assessment. Despite these limitations, this study has several strengths. Our study offers a comprehensive and updated evaluation of the inequality in GC screening, encompassing both organized and opportunistic screenings. Additionally, as we have used data from a high-quality national survey designed to monitor screening behavior, our results are highly representative and generalizable.

In conclusion, the KNCSP has played a crucial role in increasing the rate of organized screening while simultaneously reducing the prevalence of opportunistic screening. Over the 14-year study period, no socioeconomic inequalities were observed during the organized screening. Overall, our study sheds light on the positive impacts of the KNCSP and highlights the importance of addressing socioeconomic inequalities in accessing screening services. These findings have implications for improving cancer screening programs and promoting equitable healthcare delivery. Future studies should continue to monitor the issue of GC inequality carefully not only in the screening services but also a comprehensive evaluation of the cancer incidence and outcomes using additional factors besides income and education.

## Data availability statement

The datasets supporting this study’s findings are available from the corresponding author upon reasonable request.

## Ethics statement

The studies involving humans were approved by Institutional Review Board of the National Cancer Center of Korea. The studies were conducted in accordance with the local legislation and institutional requirements. The ethics committee/institutional review board waived the requirement of written informed consent for participation from the participants or the participants’ legal guardians/next of kin because All participants were informed of the purpose and use of the data before enrollment in the survey, and the requirement for written informed consent was waived.

## Author contributions

XL: Conceptualization, Formal analysis, Investigation, Methodology, Writing – original draft. KL: Formal analysis, Writing – review & editing. JJ: Methodology, Writing – review & editing. MS: Methodology, Writing – review & editing. KC: Conceptualization, Funding acquisition, Methodology, Supervision, Writing – review & editing.
