# Supplementary material for: Socioeconomic inequality in organized and opportunistic screening for gastric cancer: results from the Korean National Cancer Screening Survey 2009–2022
Source: Front Public Health. 2023 Oct 9;11:1256525. doi: 10.3389/fpubh.2023.1256525 (PMC10591186; doi:10.3389/fpubh.2023.1256525)
Supplement: Supplementary file 1 [file Table_1.docx]

Supplementary Material

**Table S1.** Baseline characteristics of the study population in the Korean National Cancer Screening Survey, 2009–2022.

|  | **2009** | **2010** | **2011** | **2012** | **2013** | **2014** | **2015** | **2016** | **2017** | **2018** | **2019** | **2020** | **2021** | **2022** |
| --- | --- | --- | --- | --- | --- | --- | --- | --- | --- | --- | --- | --- | --- | --- |
| **Total,** no. | 1,640 | 3,411 | 3,474 | 3,498 | 3,509 | 3,441 | 3,441 | 3,480 | 3,484 | 3,495 | 3,539 | 3,647 | 3,552 | 3,552 |
| **Sex,** % |  |  |  |  |  |  |  |  |  |  |  |  |  |  |
| Male | 49.2 | 49.3 | 48.8 | 49.6 | 49.5 | 50.3 | 50.3 | 49.8 | 49.8 | 49.8 | 49.3 | 49.3 | 49.9 | 49.9 |
| Female | 50.8 | 50.8 | 51.2 | 50.4 | 50.5 | 49.7 | 49.7 | 50.2 | 50.2 | 50.2 | 50.7 | 50.7 | 50.1 | 50.1 |
| **Age group,** % |  |  |  |  |  |  |  |  |  |  |  |  |  |  |
| 40–49 | 42.9 | 41.4 | 39.5 | 39.6 | 38.6 | 38.3 | 38.3 | 36.6 | 35.6 | 34.9 | 31.5 | 30.6 | 31.7 | 31.7 |
| 50–59 | 30.6 | 32.5 | 33.5 | 33.8 | 34.8 | 35.4 | 35.4 | 34.9 | 34.6 | 34.5 | 32.4 | 31.8 | 33.1 | 33.1 |
| 60–69 | 19.6 | 20.0 | 19.6 | 19.1 | 19.6 | 19.9 | 19.9 | 22.0 | 22.5 | 23.3 | 22.8 | 24.1 | 27.0 | 27.0 |
| 70–75 | 7.0 | 6.1 | 7.5 | 7.5 | 7.0 | 6.4 | 6.4 | 6.4 | 7.3 | 7.4 | 13.3 | 13.5 | 8.2 | 8.2 |
| **Residential area,** % |  |  |  |  |  |  |  |  |  |  |  |  |  |  |
| Metropolitan | 46.2 | 43.9 | 45.0 | 43.9 | 44.0 | 44.5 | 45.6 | 43.9 | 44.9 | 44.0 | 43.7 | 46.7 | 43.0 | 43.0 |
| Non-metropolitan | 53.8 | 56.1 | 55.0 | 56.1 | 56.0 | 55.5 | 54.4 | 56.2 | 55.1 | 56.0 | 56.3 | 53.3 | 57.0 | 57.0 |
| **Education level,** % |  |  |  |  |  |  |  |  |  |  |  |  |  |  |
| Elementary or lower | 18.1 | 9.6 | 9.8 | 11.4 | 6.0 | 6.4 | 5.6 | 4.4 | 5.0 | 4.2 | 4.6 | 4.7 | 3.8 | 3.3 |
| Middle school graduates | 13.3 | 12.9 | 12.7 | 10.2 | 8.4 | 9.4 | 9.4 | 7.2 | 11.0 | 9.6 | 11.9 | 10.1 | 7.8 | 7.2 |
| High school graduates | 46.7 | 52.6 | 52.7 | 53.2 | 53.9 | 56.2 | 54.9 | 55.6 | 52.9 | 51.9 | 52.3 | 53.5 | 50.2 | 49.5 |
| College/University or higher | 22.0 | 25.0 | 24.8 | 25.2 | 31.7 | 28.0 | 30.1 | 32.8 | 31.1 | 34.4 | 31.3 | 31.7 | 38.2 | 40.0 |
| **Household income,** % |  |  |  |  |  |  |  |  |  |  |  |  |  |  |
| Low | 28.9 | 32.5 | 32.8 | 28.0 | 25.4 | 36.8 | 37.1 | 37.7 | 38.3 | 35.5 | 34.9 | 36.6 | 30.2 | 25.2 |
| Middle | 45.1 | 36.4 | 36.4 | 44.8 | 34.6 | 26.5 | 27.7 | 30.3 | 31.7 | 26.0 | 37.4 | 32.5 | 32.6 | 28.6 |
| High | 26.0 | 31.1 | 30.9 | 27.2 | 40.0 | 36.7 | 35.3 | 32.0 | 30.0 | 38.5 | 27.7 | 31.0 | 37.1 | 46.2 |
| Note: The number is presented as unweighted frequency. | | | | | | | | | | | | | | |
