# Supplementary material for: Socioeconomic inequality in organized and opportunistic screening for gastric cancer: results from the Korean National Cancer Screening Survey 2009–2022
Source: Front Public Health. 2023 Oct 9;11:1256525. doi: 10.3389/fpubh.2023.1256525 (PMC10591186; doi:10.3389/fpubh.2023.1256525)
Supplement: Supplementary file 2 [file Table_2.docx]

Supplementary Material

**Table S2.** Overall screening rates for gastric cancer according to socioeconomic status in the Korean National Cancer Screening Survey 2009–2022 (%).

|  | **2009** | **2010** | **2011** | **2012** | **2013** | **2014** | **2015** | **2016** | **2017** | **2018** | **2019** | **2020** | **2021** | **2022** | **AAPC (95% CI)** | |
| --- | --- | --- | --- | --- | --- | --- | --- | --- | --- | --- | --- | --- | --- | --- | --- | --- |
| **Total** | 57.0 | 65.0 | 64.6 | 70.9 | 73.6 | 76.7 | 74.8 | 73.0 | 72.2 | 72.8 | 70.8 | 68.9 | 76.6 | 75.2 | | 1.9 (0.8–3.0) |
| **Sex** |  |  |  |  |  |  |  |  |  |  |  |  |  |  | |  |
| Male | 52.4 | 63.6 | 63.5 | 69.9 | 72.4 | 77.2 | 75.4 | 71.5 | 70.4 | 73.1 | 70.8 | 69.3 | 76.8 | 76.4 | | 2.2 (0.2–4.3) |
| Female | 61.5 | 66.4 | 65.6 | 71.9 | 74.8 | 76.2 | 74.2 | 74.5 | 73.9 | 72.5 | 70.8 | 68.6 | 76.5 | 74.1 | | 1.3 (-0.1–2.6) |
| **Age group** |  |  |  |  |  |  |  |  |  |  |  |  |  |  | |  |
| 40–49 | 47.5 | 59.7 | 59.0 | 65.1 | 72.6 | 75.9 | 70.7 | 68.6 | 69.8 | 72.1 | 70.0 | 66.0 | 74.0 | 72.5 | | 2.5 (0.5–4.4) |
| 50–59 | 62.5 | 69.5 | 68.8 | 76.2 | 74.6 | 77.5 | 77.4 | 74.4 | 75.0 | 74.4 | 74.1 | 71.3 | 79.9 | 76.9 | | 0.6 (-0.1–1.3) |
| 60–69 | 69.8 | 69.8 | 68.9 | 74.9 | 74.4 | 78.0 | 79.4 | 76.5 | 71.9 | 74.5 | 75.1 | 73.6 | 76.2 | 77.8 | | 1.0 (-0.5–2.5) |
| 70–75 | 56.1 | 62.1 | 63.6 | 67.6 | 72.2 | 73.4 | 71.0 | 77.9 | 70.8 | 63.5 | 56.8 | 61.8 | 74.8 | 70.5 | | 1.8 (-2.8–6.5) |
| **Residential area** |  |  |  |  |  |  |  |  |  |  |  |  |  |  | |  |
| Metropolitan | 55.0 | 65.1 | 64.3 | 69.4 | 74.3 | 78.6 | 75.0 | 75.5 | 73.3 | 76.4 | 73.0 | 68.3 | 77.2 | 77.2 | | 2.2 (0.7–3.8) |
| Non-metropolitan | 58.8 | 65.0 | 64.7 | 72.0 | 73.1 | 75.1 | 74.7 | 71.0 | 71.3 | 70.0 | 69.0 | 69.5 | 76.2 | 73.8 | | 1.6 (-0.1–3.4) |
| **Education level** |  |  |  |  |  |  |  |  |  |  |  |  |  |  | |  |
| Elementary or lower | 63.6 | 65.4 | 65.2 | 70.3 | 75.0 | 70.6 | 63.5 | 79.4 | 81.7 | 52.2 | 55.8 | 61.0 | 71.6 | 66.3 | | 0.3 (-1.5–2.2) |
| Middle school graduates | 64.2 | 65.1 | 64.2 | 72.8 | 69.9 | 75.0 | 74.6 | 80.1 | 66.9 | 73.1 | 56.2 | 64.0 | 71.8 | 68.7 | | 0.1 (-1.4–1.5) |
| High school graduates | 52.5 | 63.3 | 62.9 | 71.0 | 74.4 | 77.2 | 75.9 | 72.8 | 70.7 | 72.5 | 72.2 | 70.6 | 75.9 | 73.4 | | 1.9 (0.4–3.5) |
| College or higher | 56.9 | 68.4 | 67.9 | 70.0 | 73.0 | 77.5 | 75.1 | 70.9 | 74.9 | 75.7 | 76.0 | 68.9 | 79.1 | 79.5 | | 1.1 (0.4–1.8) |
| **Household income** |  |  |  |  |  |  |  |  |  |  |  |  |  |  | |  |
| Low | 60.3 | 64.2 | 63.3 | 69.7 | 71.1 | 75.0 | 71.8 | 73.6 | 69.6 | 68.5 | 65.6 | 66.6 | 72.3 | 72.1 | | 1.5 (0.0–3.0) |
| Middle | 53.8 | 61.5 | 61.3 | 69.1 | 71.9 | 78.9 | 73.3 | 72.2 | 70.4 | 73.7 | 69.2 | 67.0 | 76.4 | 73.8 | | 2.4 (0.9–4.0) |
| High | 59.0 | 69.9 | 69.6 | 74.9 | 76.7 | 76.8 | 79.3 | 73.0 | 77.2 | 76.2 | 79.4 | 73.8 | 80.3 | 77.8 | | 0.8 (0.1–1.4) |
| Screening with recommendation was defined as the upper gastrointestinal series, or upper endoscopy, during the past two years.  AAPC, Average Annual Percent Change; 95% CI, 95% confidence interval. | | | | | | | | | | | | | | | | |
