# Supplementary figures and images for: Socioeconomic inequality in organized and opportunistic screening for gastric cancer: results from the Korean National Cancer Screening Survey 2009–2022
Source: Front Public Health. 2023 Oct 9;11:1256525. doi: 10.3389/fpubh.2023.1256525 (PMC10591186; doi:10.3389/fpubh.2023.1256525)

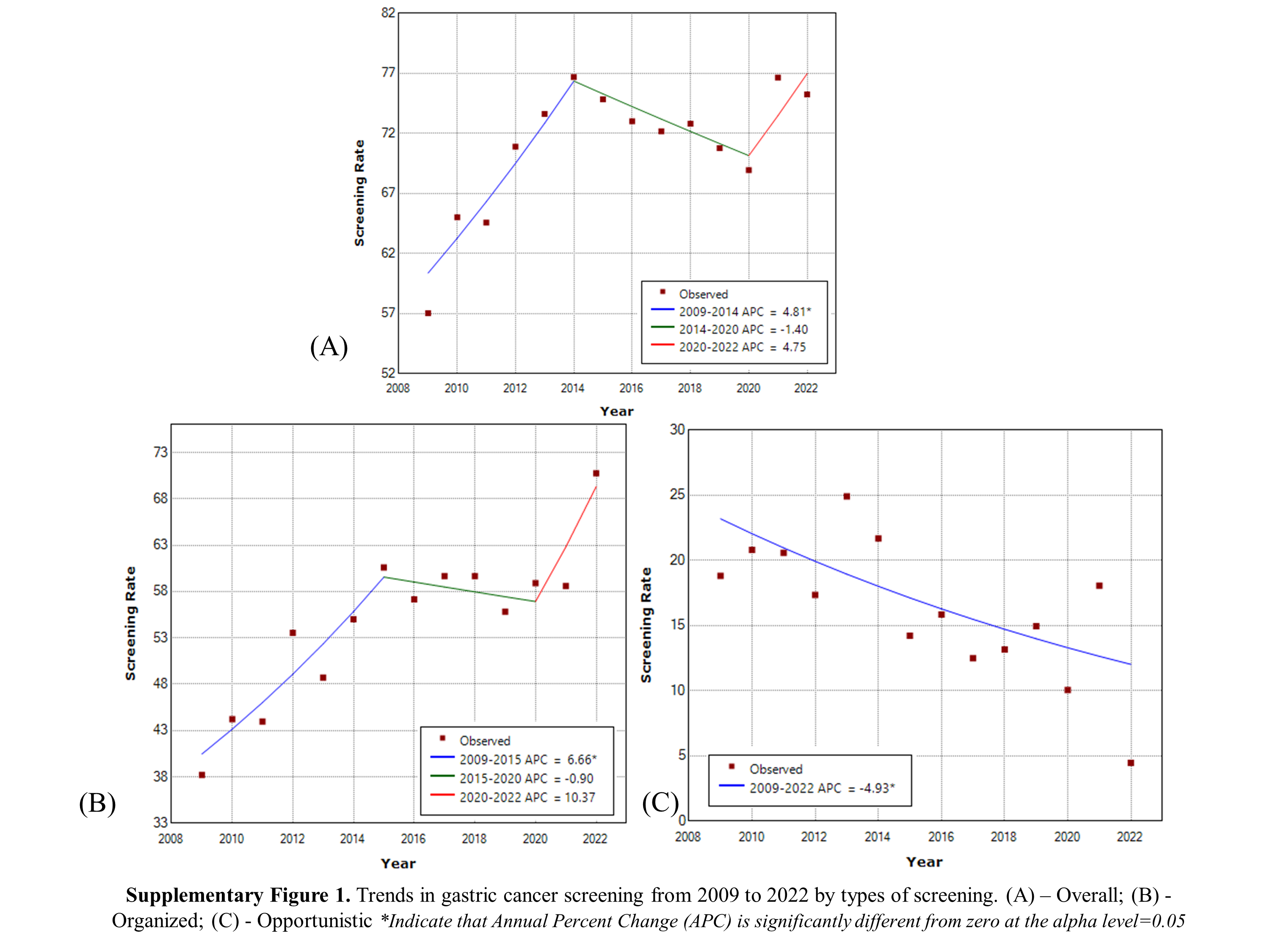

Supplement: Supplementary file 3 [file Image_1.TIF]

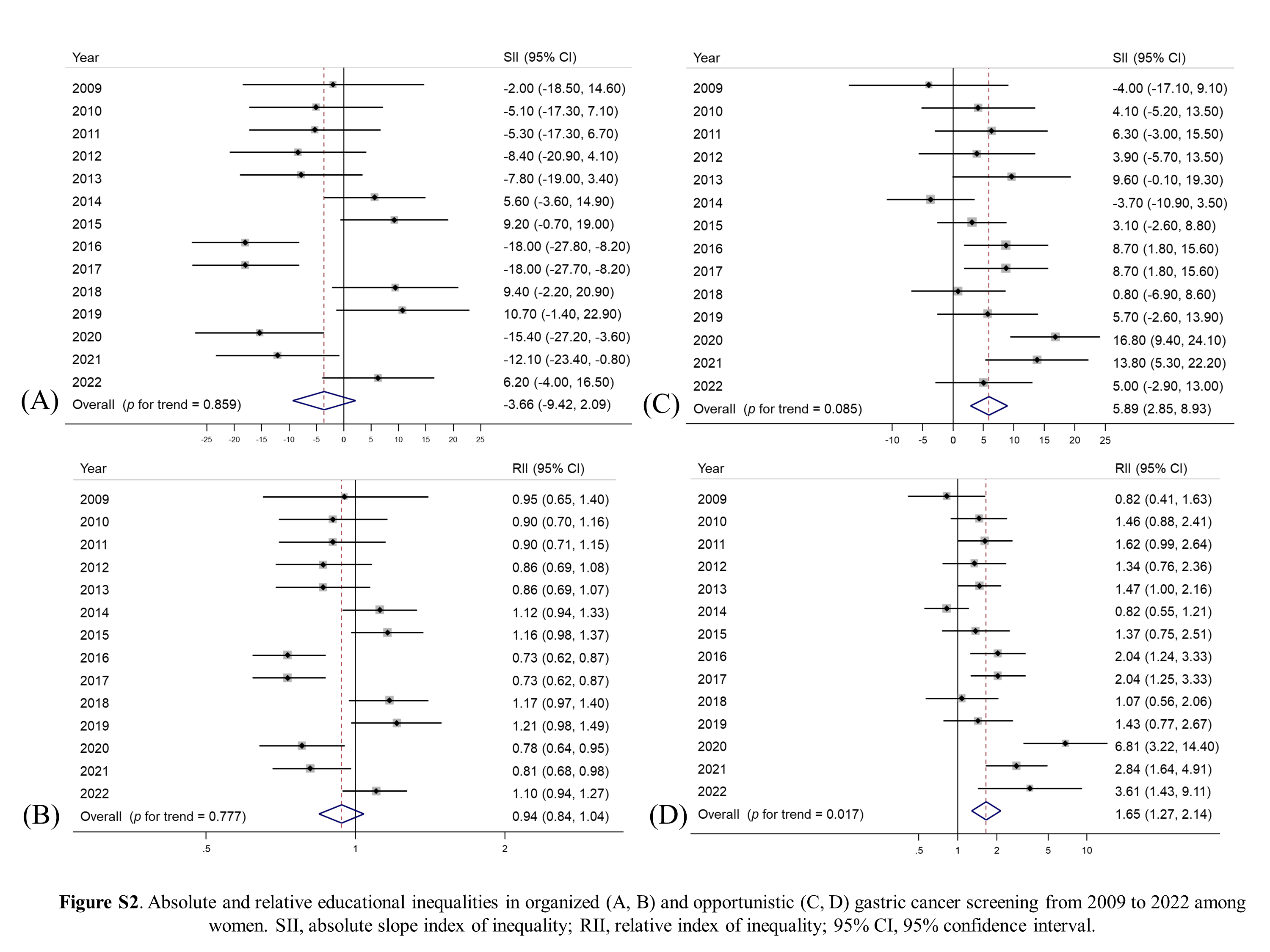

Supplement: Supplementary file 4 [file Image_2.TIF]

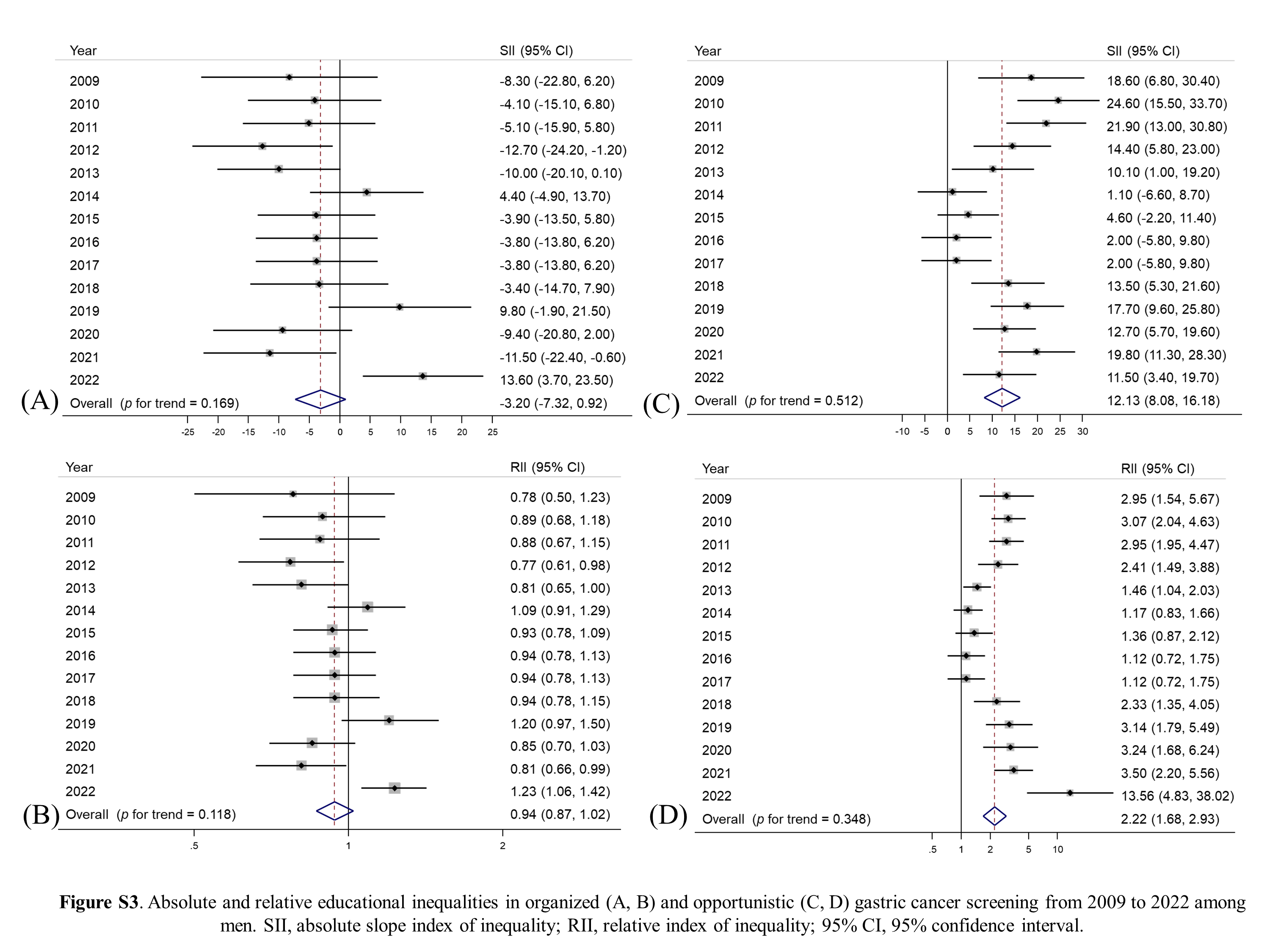

Supplement: Supplementary file 5 [file Image_3.TIF]

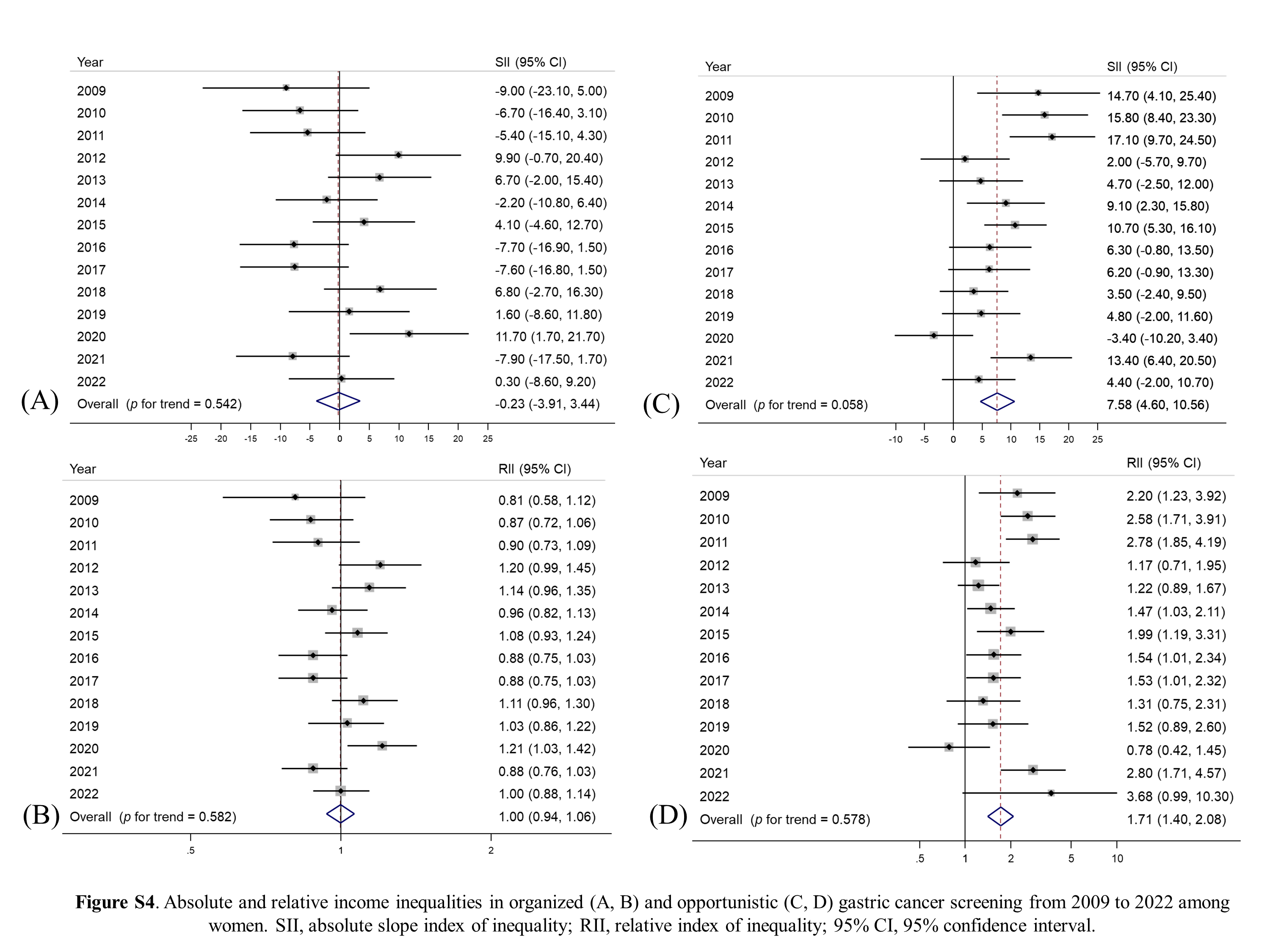

Supplement: Supplementary file 6 [file Image_4.TIF]

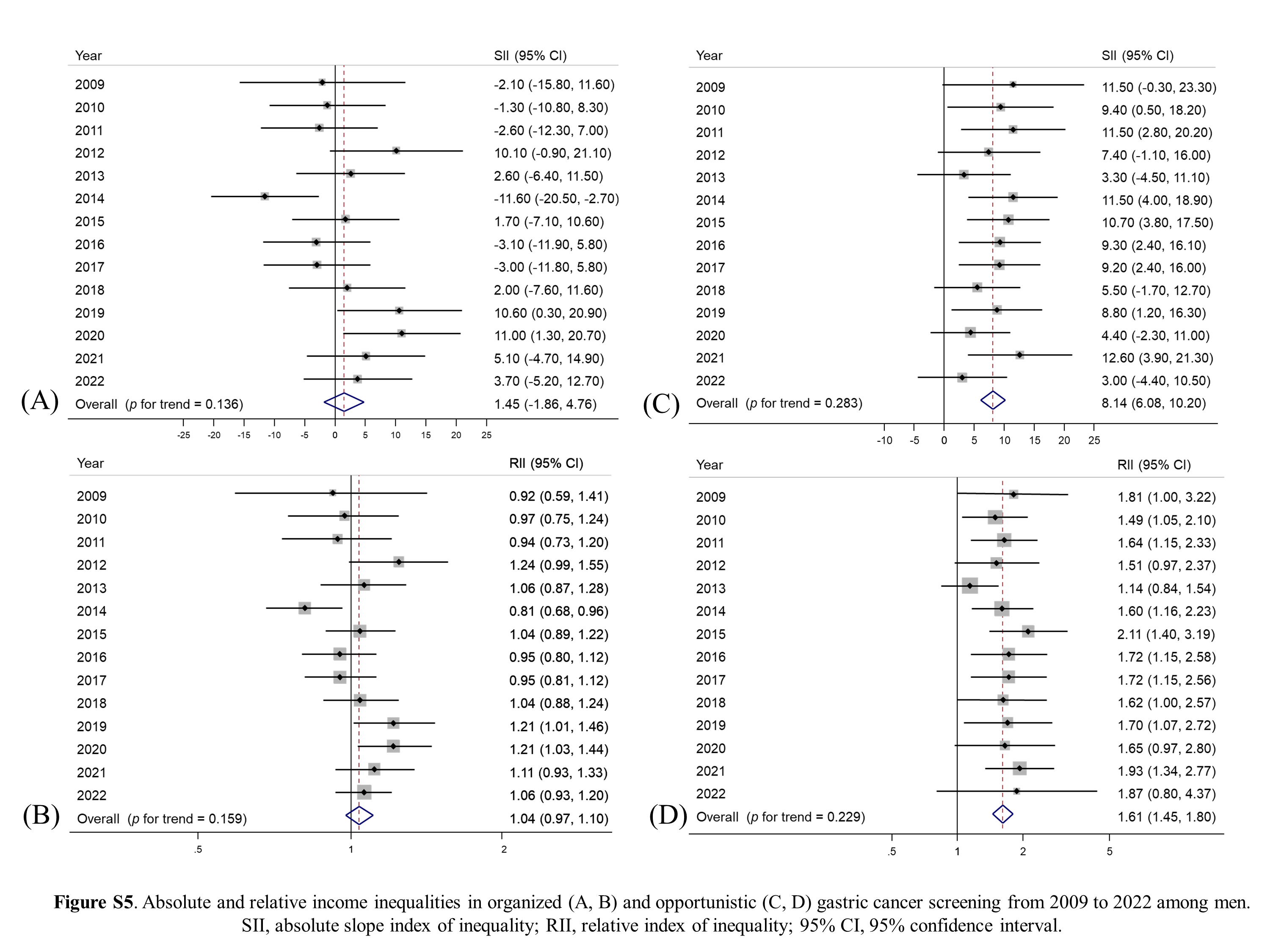

Supplement: Supplementary file 7 [file Image_5.TIF]

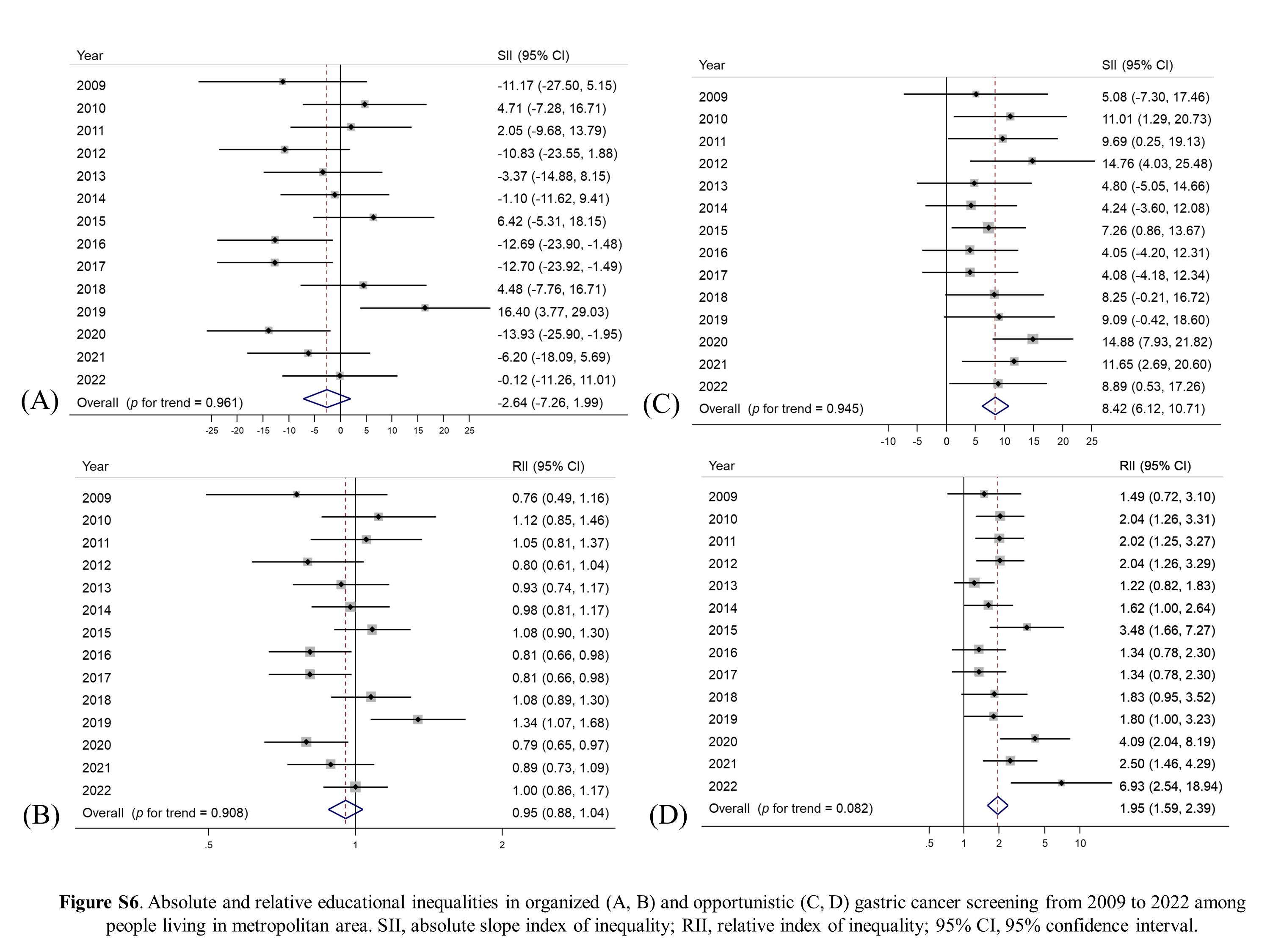

Supplement: Supplementary file 8 [file Image_6.TIF]

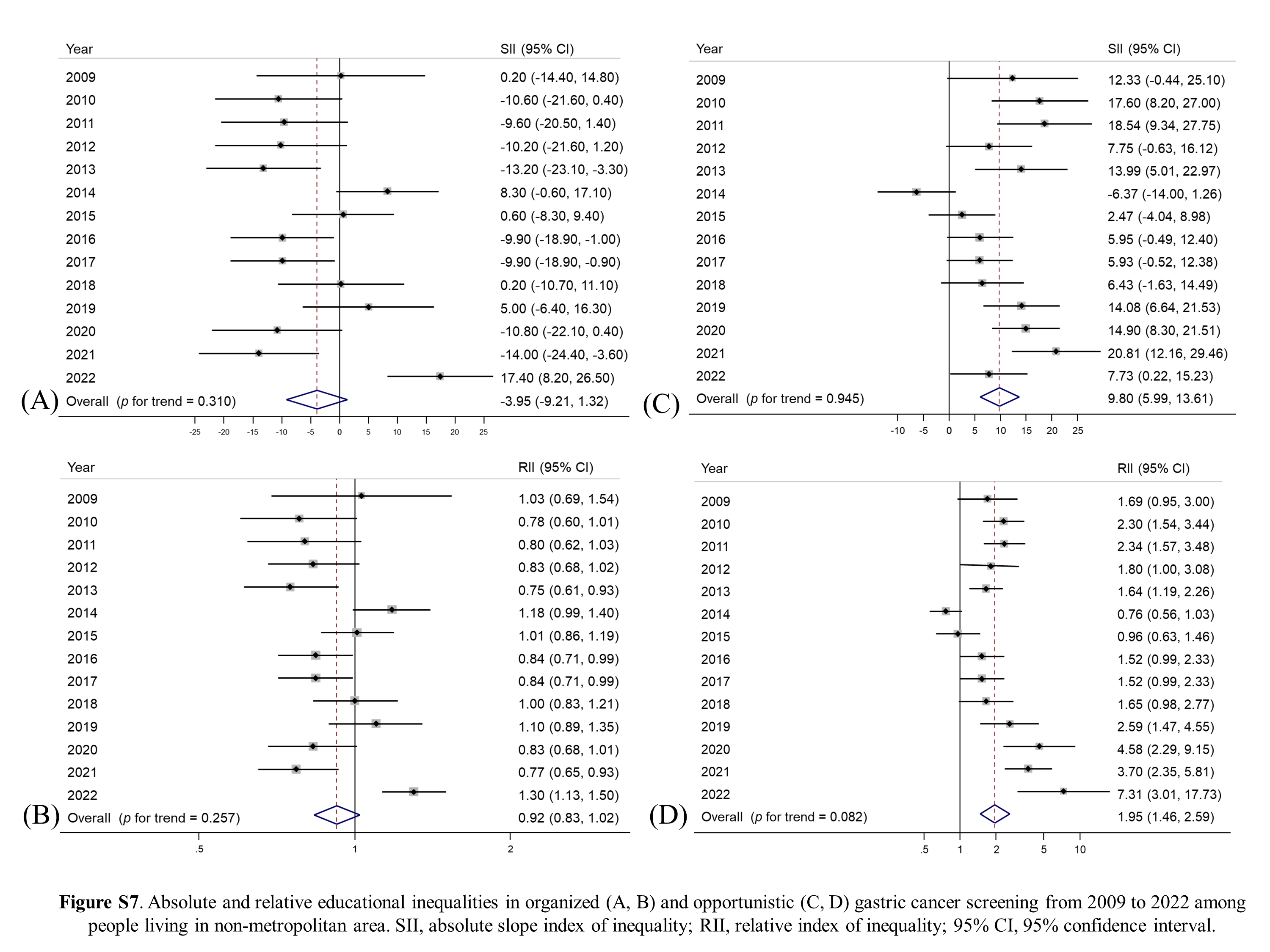

Supplement: Supplementary file 9 [file Image_7.TIF]

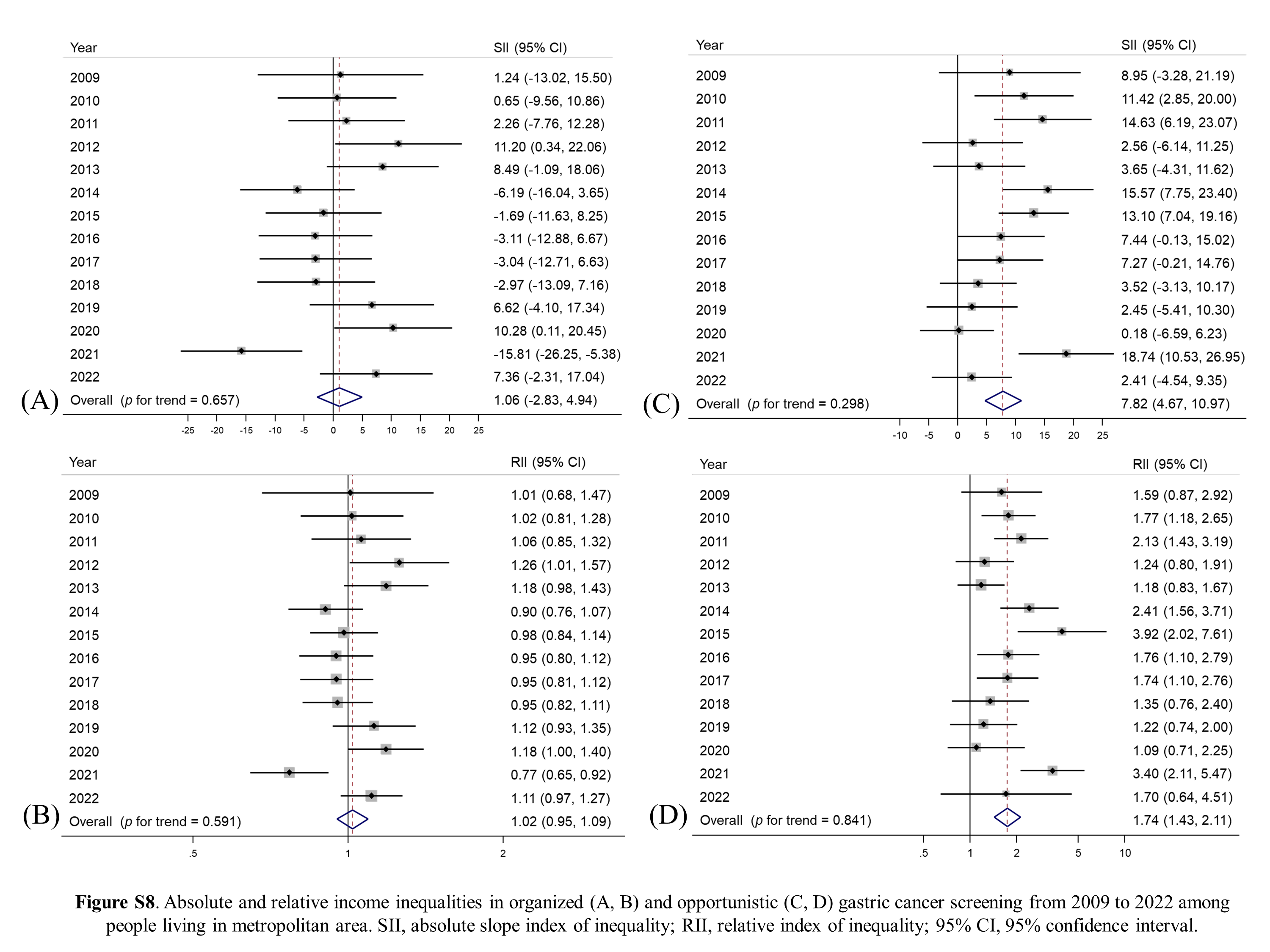

Supplement: Supplementary file 10 [file Image_8.TIF]

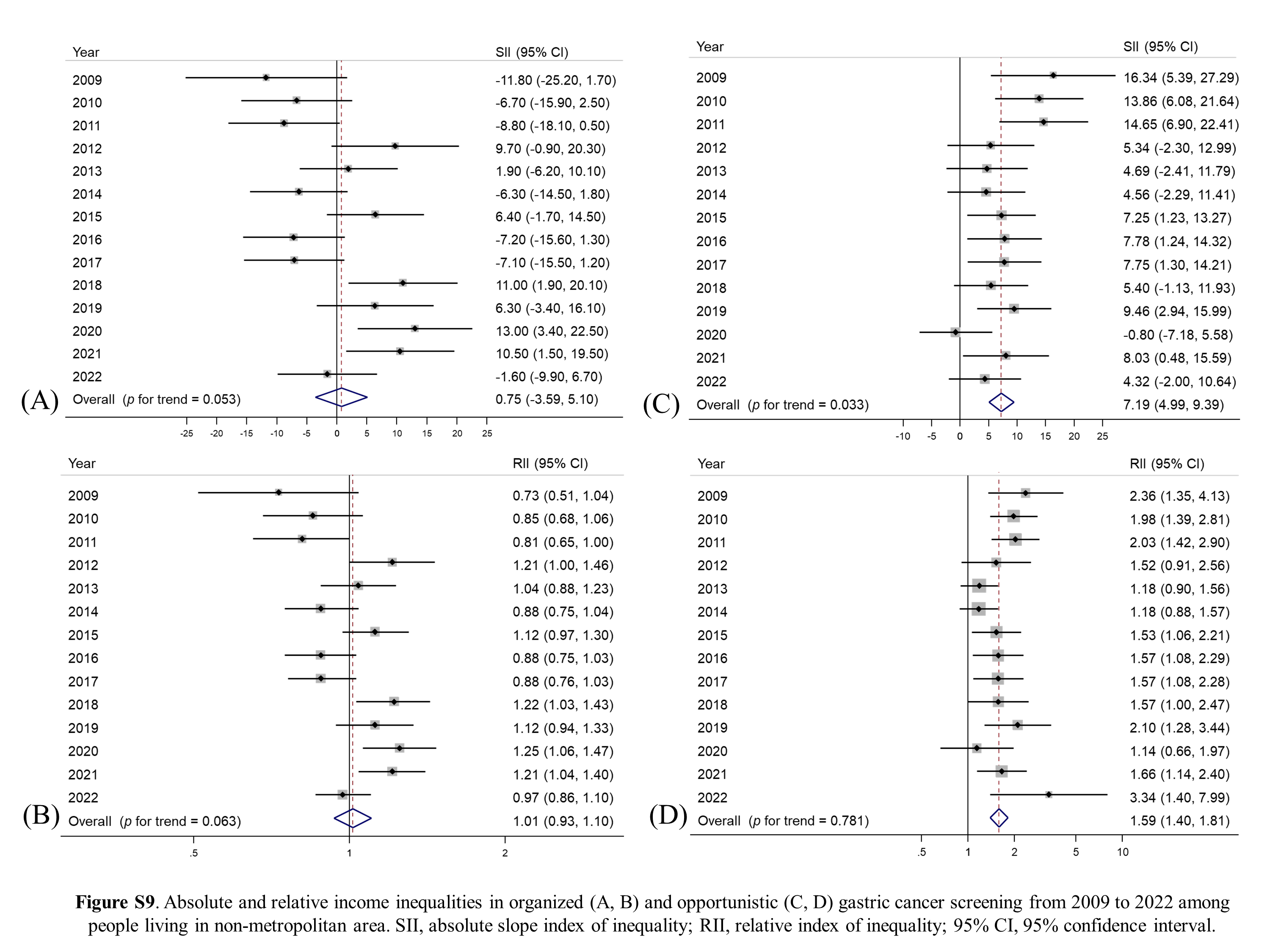

Supplement: Supplementary file 11 [file Image_9.TIF]
